# Polymorphic microsatellites in the human bloodfluke, *Schistosoma japonicum*, identified using a genomic resource

**DOI:** 10.1186/1756-3305-4-13

**Published:** 2011-02-07

**Authors:** Ning Xiao, Justin Remais, Paul J Brindley, Dongchuan Qiu, Robert Spear, Yang Lei, David Blair

**Affiliations:** 1 Institute of Parasitic Diseases, Sichuan Center for Disease Control and Prevention, Chengdu, Sichuan 610041, PR China; 2 Department of Environmental Health, Rollins School of Public Health, Emory University, 1518 Clifton Rd. NE, Atlanta, Georgia 30322, USA; 3 Department of Microbiology, Immunology & Tropical Medicine, George Washington University Medical Center, 2300 Eye Street, NW, Washington, DC 20037, USA; 4 Department of Environmental Health Sciences, School of Public Health, 50 University Hall, University of California, Berkeley, CA 94720, USA; 5 School of Marine and Tropical Biology, James Cook University, Townsville, Queensland, 4811, Australia

## Abstract

Re-emergence of schistosomiasis in regions of China where control programs have ceased requires development of molecular-genetic tools to track gene flow and assess genetic diversity of *Schistosoma *populations. We identified many microsatellite loci in the draft genome of *Schistosoma japonicum *using defined search criteria and selected a subset for further analysis. From an initial panel of 50 loci, 20 new microsatellites were selected for eventual optimization and application to a panel of worms from endemic areas. All but one of the selected microsatellites contain simple tri-nucleotide repeats. Moderate to high levels of polymorphism were detected. Numbers of alleles ranged from 6 to 14 and observed heterozygosity was always >0.6. The loci reported here will facilitate high resolution population-genetic studies on schistosomes in re-emergent foci.

## Findings

The Asian bloodfluke, *Schistosoma japonicum*, causes serious human disease in several parts of eastern Asia, and in particular in China where more than 30 million people living in the tropical and subtropical zones are at risk [1]. On the heels of widespread progress in controlling *S. japonicum *over the past two decades [2], China is facing the challenges posed by re-emergence of schistosomiasis in localities where control activities have nearly ceased and where apparent elimination had been achieved [2,3]. In comparison to traditional assessment methods, molecular tools will be increasingly important as China targets regions with low prevalence and low infection intensity for elimination [4-6] on the path to reducing schistosome infection to less than 1% by 2015 [2]. Such tools will be important for high-resolution monitoring of infections in snails and in mammalian hosts, elucidating transmission networks, and improving the targeting of interventions to achieve final elimination of the disease.

This important pathogen has already received attention from population geneticists using microsatellites [7,8]. Most of the loci used had been found by examination of the GenBank accessions for *S. japonicum *data existing at that time or through testing of primers that amplified microsatellite loci in another bloodfluke, *S. mansoni*. A near-complete draft genome of *S. japonicum *has been released by the Chinese Human Genome Center in Shanghai [9] and the raw data are available at http://lifecenter.sgst.cn/schistosoma/cn/schistosomaCnIndexPage.do. An earlier study [10] reported 17 loci from this resource. In this study we report on additional loci suitable for research into gene flow in *S. japonicum *in Sichuan Province. Results of this search are presented here. We searched this resource for microsatellite loci using the following criteria:

• Repeats should be 2, 3 or 4-mer,

• The number of tandem repeats should be in the range 10-25,

• Loci should not include compound repeats,

• Loci should be flanked by single-copy DNA sequences to which PCR primers could be targeted,

• Loci should be far apart so as to avoid linkage disequilibrium. In practice, since chromosomal assignment of super-contigs is unknown as yet, loci were selected from different super-contigs,

• Loci should not be in, or close to, known or predicted coding regions.

The search was undertaken using the software SciRoKo [11]. Following the above criteria, we identified 72 new loci for which primers were designed and synthesized. For 30 loci showing a single band of the correct size in a preliminary screening using an agarose gel, new forward primers were synthesized to permit M13 tailing [12,13]. All 30 loci were then used for genotyping of ten adult worms. Loci failing to provide clear signals in the expected size range, or that lacked polymorphism, were not considered further. Finally 20 new loci, plus two from [10], were optimized for PCR and used in the genotyping of 20 individual adult worms. Primer annealing temperatures were designed to be very similar with eventual multiplexing in mind.

The Sichuan Center for Disease Control and Prevention (SCDC) maintains *S. japonicum *lifecycles, including *S. japonicum *from infected snails sourced from Hubei and Anhui Provinces passed through rabbit hosts. For this study, existing samples were obtained from SCDC of adult schistosomes that were derived from a single passage of mixed Hubei and Anhui cercariae through a definitive host. Genomic DNA was extracted from 10 individual male and 10 female worms by incubation in hot sodium hydroxide with pH adjustment using a Tris solution (HotSHOT) [14]. The lysates were used as templates for PCR directly. Each worm was genotyped individually.

All PCRs were carried out in a 10 μl reaction mixture containing 0.5 μl of template DNA (about 17.5 ng), 0.5 μM of each primer but 0.125 μM of any forward primer with an M13 tail, and 5 μl of 2X GoTaq Green Master Mix (Promega Corporation, WI, USA). For PCR amplification, templates were denatured at 94°C for 5 minutes followed by 30 cycles (94°C 30 seconds, 55°C 45 seconds, 72°C 45 seconds), and then by 8 cycles ( 94°C 30 seconds, 53°C 45 seconds, 72°C 45 seconds), and a final extension at 72°C for 10 minutes. PCR products were separated using an ABI 3130 XL automated DNA sequencer with ABI GS500 LIZ internal size standards. Results were read in GeneMapper 4.0 software (Applied Biosystems).

Estimates of heterozygosity were made, tests conducted for Hardy-Weinberg equilibrium and linkage disequilibrium, and alleles counted. The software package GenAlEx [15] was used for most data analysis. Micro-Checker [16] was used to identify loci at which null (non-amplifying) alleles might be present. Use of the Bonferroni-adjusted 95% confidence interval indicates that null alleles may occur only at locus SjP9, one of the loci reported by [10].

Table 1 presents the findings for each locus, including numbers of alleles and observed heterozygosity (*H*). Observed heterozygosity (*Ho*) at all loci was high, never below 0.6. A few loci deviated significantly from Hardy Weinberg expectations (Table 1), including SjP9 at which null alleles were suspected. Surprisingly, many pairs of loci were in linkage disequilibrium (data not shown). We consider this to be a consequence of the fact that adult worms were derived from pooled cercariae from infected snails: each snail is likely to yield many sibling/clonal cercariae, resulting in significant linkage disequilibrium (e.g. [10]). The pooling of cercariae from two distinct populations is likely to increase this effect. Previous workers [7] noted a high frequency of linkage disequilibrium in *S. japonicum *and considered it due to inbreeding and non-random mating.

**Table 1:** Primer sequences and other characteristics for each of 22 microsatellite loci amplified from 20 specimens of *Schistosoma japonicum*

**Locus name**	**Primer sequences** (**tails removed**)	Repeat unit	Length of locus in Sj Genome draft (excl tails)	Length range (PCR product - bp excl tail)	No. of alleles	In HWE?	Ho	He
SjP4	F:ACAAGCTCCAATCGTCTCTGA R:GAATACTGCCGCCCTTGTAA	TAA	217	182-244	14		0.789	0.831

SjP9	F:GATGAAACAGATACCCAGCAC R:TGCATGTAAAAATGGCTTGC	TAA	283	239-301	14	**	0.600	0.915

SjP18	F:TCCTTTATCTGGGCTGTGGA R:TTTCAGCAGGATAACATGACG	TGA	286	261-298	7		0.684	0.703

SjP19	F:GGTATCTTCGCTTTTTAGCATGG R:TCCTAGGGTGTGGTATCAGAG	ATT	196	161-257	12	**	0.737	0.896

SjP22	F:CAAAGCCTAAACGTCATAGACAG R:CAACCACCGATAAGTAGAGTGGA	TTA	150	105-167	11	*	0.850	0.892

SjP23	F:GTACGATATGAGGGAAAGTTCA R:CTCTCCTTCAGACGAATTGAG	TAA	219	192-253	14		1.00	0.933

SjP26	F:CAAGGGAACATTGTACATGAAG R:TGGTAAAGGAGAAAGTGAACG	TAA	307	229-312	7		0.700	0.659

SjP28	F:TAACGCCTTTTCCCACATTC R:ATAACCACGATGGGAACCAA	TTA	242	232-269	13	**	0.900	0.926

SjP32	F:TGTCACCGAGTCTTCATTAGC R:ACAGTCAGTAGACCTGGATAAAC	TTA	175	142-192	14		0.950	0.929

SjP34	F:GGCGACCATACATAAGGAGAAT R:GACCGATTTCTAATGGAGCA	TAA	409	381-430	13	*	0.778	0.908

SjP37	F:TCCTTGACACGAGGTACATGT R:ATTACGTAACAGAAGGCTGGA	TAT	290	240-314	8		0.833	0.714

SjP39	F:GACGACTGTTAAGTCCATCTGA R:ATAACCAATCTCCACGAAAGC	ATT	239	223-251	8		0.900	0.871

SjP42	F:GCTGCAGCTTCTGTGTAGTAA R:GTCTTGCTCAGATCAGTTCGT	TAA	216	199-234	9		0.950	0.855

SjP43	F:ACAATGGCTATTGGTTGAGTAG R:GGAGCATGCGTATATGGAAAA	TAA	188	184-235	9		0.750	0.854

SjP45	F:ATAACACCGAATCTGTTCAGC R:TAATCCGGTCAGGATGTATGT	TAT	156	150-244	13		0.850	0.921

SjP48	F:TTGTTGGGTAGTGATGGTAGG R:TAGTTCATTCCACCTCTTGGA	TAA	246	251-273	6		0.650	0.795

SjP54	F:TTAGGCTTGTTGGTGCTGATA R:AGGTAAAGCAAATCCCATAGC	GGTA	437	373-446	9		1.00	0.712

SjP58	F:TCCCAGTACCAATGTAGATGTG R:CTAATAAAGTCGTCAAGGAGCA	AAT	227	439-499	12	**	0.800	0.921

SjP60	F:CGATTCATTCATAGCCTGACT R:GAATCCCATCACAGATTAACG	TAT	155	134-165	10		0.900	0.867

SjP61	F:TCATCTTGTCACCAACTAGGC R:GCTTGGAGGAGGCTAAAATAC	TAT	188	158-238	9		0.700	0.679

SjP63	F:ACCGCCACTACCACTAACCTCA R:TTGACCTGAAATCTGTCCCTA	TAA	390	333-389	13	**	0.800	0.904

SjP88	F:GCTTTCCAGGCATAAACTTCAC R:TCTCCTAATGATGGGAACAG	TAA	408	380-417	9		0.950	0.887


Sequences of microsatellites reported here have been deposited in GenBank [AB604199 - AB604218].

Note that the first two loci are from [10] and are included for comparative purposes.

The annealing temperature of PCRs for all loci is 55°C

* P < 0.05 ** P < 0.01 as determined in GenAlEx.

The loci presented here are likely to be specific for *S. japonicum*. Blast searches of the draft genome of *S. mansoni *(http://www.sanger.ac.uk/Projects/S_mansoni/) failed to find any matches that would indicate conservation of flanking regions in both species (not shown). Searches of the draft genome of *S. japonicum *yielded only a single "hit" for each locus.

The microsatellite loci reported here are, with one exception (SjP54), perfect trinucleotide repeats, making scoring easier than for dinucleotide and/or compound repeats. The diversity of alleles and genotypes present in the populations we sampled demonstrate the utility of these markers for future studies on epidemiology of *S. japonicum *in eastern Asia. Finally, the obvious genetic diversity within field populations of *S. japonicum *in China demonstrated by these polymorphic microsatellite loci confirms the recent report of marked genetic diversity in this parasite detected by analysis of the *S. japonicum *transcriptome and proteome [4].

## Authors' contributions

NX, JR, PB and DB conceived of and designed the study, and were assisted by DQ and RS who coordinated the study. NX and YL carried out the molecular genetic studies, and were assisted by JR, PB and DB in analysing the results. NX, JR, PB and DB drafted the manuscript. All authors read and approved the final manuscript.

## Competing interests

The authors declare that they have no competing interests.
